# A tale of two public dental benefit programs: Iowa dentist participation in traditional Medicaid versus a Medicaid expansion program

**DOI:** 10.1186/s12903-019-0771-z

**Published:** 2019-05-24

**Authors:** Julie C. Reynolds, Susan C. McKernan, Peter C. Damiano, Raymond A. Kuthy

**Affiliations:** 1 University of Iowa Public Policy Center, Iowa City, IA USA; 2 University of Iowa College of Dentistry, Iowa City, IA USA

**Keywords:** Access to care, Dental benefits, Dental health services, Dental insurance, Dental private practice, Health policy, Managed care programs, Medicaid, Public health/Community dentistry

## Abstract

**Background:**

In Iowa from 2014 to 2017, there were 2 separate public dental benefit programs for Medicaid-enrolled adults: one for the Medicaid expansion population called the Dental Wellness Plan (DWP), and one for the traditional, non-expansion adult Medicaid population. The programs differed with respect to reimbursement, administration, and benefit structure. This study explored differences in patterns and predictors of dentist participation in the two programs.

**Methods:**

Authors sent a survey to all private practice dentists in Iowa (*n* = 1301) 2 years after DWP implementation. Descriptive, bivariate, and logistic regression analyses were used to examine patterns and predictors of dentist participation in Medicaid and DWP.

**Results:**

Overall rates of dentists’ acceptance of new Medicaid and DWP patients were 45 and 43%, respectively. However, Medicaid participants were much more likely than DWP participants to place limits on patient acceptance. Adjusting for other factors, practice busyness was the only significant predictor of DWP participation, and practice location was the only significant predictor of Medicaid participation. Dentists who were not busy enough were more than twice as likely to participate in DWP compared to others, and dentists in rural areas were almost twice as likely to participate in Medicaid compared to dentists in urban areas.

**Conclusions:**

Dentist participation in Medicaid is an ongoing concern for states aiming to ensure access to dental care for low-income populations. We found distinct participation patterns and predictors between a traditional Medicaid dental program and the DWP, suggesting different motivations for participation between the two programs.

**Electronic supplementary material:**

The online version of this article (10.1186/s12903-019-0771-z) contains supplementary material, which is available to authorized users.

## Background

Access to dental care for adults in the U.S. is an increasing concern, especially for those who have low income. While dental utilization among children and seniors has increased in the past decade, utilization among adults has declined [1]. Adults are more likely than children or seniors to experience financial barriers to care [2]. These financial barriers are largely related to state-by-state variability in dental coverage for adult Medicaid enrollees. Through the expansion of the Medicaid program to low-income adults previously ineligible for Medicaid, the Affordable Care Act (ACA) made strides to address financial barriers to care for low-income adults. Prior to the ACA, low-income adults were only eligible for Medicaid if their income was at or below 133% of the Federal Poverty Level (FPL) and they were “categorically eligible” (e.g., pregnant, elderly, disabled, or had young children); whereas the ACA expanded eligibility to *all* adults with income at or below 133% FPL regardless of categorical eligibility. However, expansion-related improvements in access to dental care were dependent on states’ decisions to: 1) expand their Medicaid program, 2) include dental benefits for Medicaid-enrolled adults, and 3) extend those dental benefits to individuals in the Medicaid expansion population.

As of January 2018, Iowa was one of 14 states that both expanded its Medicaid program and provided adult dental benefits to its traditional Medicaid adult population (i.e., those eligible for Medicaid under pre-ACA guidelines) as well as the Medicaid expansion population (i.e., adults newly eligible for Medicaid post-ACA expansion) [3]. However, from 2014 to 2017, benefits for these two populations were provided via 2 separate public dental benefit programs. Both before and after Iowa’s expansion of the Medicaid dental program in 2014, individuals in traditional Medicaid received comprehensive fee-for-service dental coverage upon enrollment, and the program was administered by the state Medicaid program. Reimbursement rates to providers in this program were approximately 40% of the usual, customary, and reasonable (UCR) fee for dental services in Iowa [4]. In contrast, adults in the Medicaid expansion population were enrolled in a separate program, called the Dental Wellness Plan (DWP). In this program, members received limited dental benefits upon enrollment with additional benefits earned by returning for regular recall exams. These levels of benefits were organized into 3 ‘tiers’ of coverage: core benefits (preventive, emergency, and stabilization services only), enhanced benefits (core + restorative, periodontal, and endodontic services), and enhanced plus benefits (enhanced + fixed and removable prosthodontic services) [5]. The program was administered by a private dental carrier, and provider reimbursement was approximately 55–60% UCR.

Access to dental care among Medicaid enrollees is heavily dependent on rates of dentist participation in Medicaid, which vary considerably by state [6]. However, dentists cite numerous barriers to treating Medicaid patients, including low reimbursement rates and high administrative burden [7, 8]. Since the DWP was designed to overcome some of these barriers with higher reimbursement rates and administration by a private dental carrier, determining how these changes affected dentist participation in these 2 public dental benefit programs is very relevant to Medicaid programs nationally. Thus the aim of this study was to explore patterns of dentist participation as well as differences in the predictors of participation in Iowa’s Medicaid expansion dental program, the DWP, compared to the traditional adult Medicaid program.

## Methods

We sent a mixed-mode survey to all private practice dentists in Iowa (*n* = 1301) in October 2016, 2 years after the DWP was implemented. Dentists received a paper survey by mail and were given the option to complete it online. We sent a reminder postcard 1 week after the initial mailing, and a second survey 2 weeks later to those who had not yet returned the survey. We obtained dentist mailing addresses, demographic data, and practice information from the Iowa Dentist Tracking System (IDTS), which has tracked information about Iowa dentists every 6 months since 1997 [9].

Survey development included the use of original survey items as well as items adapted from previous surveys, such as a previous survey of Iowa dentists about Medicaid participation [10], a 2011 survey to mental health providers in Maryland [11], and a 2011 survey of primary care providers in Washington State [12]. The complete survey instrument is provided as an Additional file 1.

We evaluated 2 dependent variables for this study: participation in DWP, and participation in Medicaid. Program participation was measured by asking survey respondents whether they currently accept new patients enrolled in DWP (yes/no), and whether they currently accept new patients enrolled in traditional Medicaid (yes/no). Dentists who indicated that they accept new patients from each program were defined as active participants in that program. Additionally, dentists who answered yes were also asked whether they accept all new patients who contact their office, or whether they place limits on acceptance of patients in each program.

We considered demographic and practice characteristics as potential independent variables associated with dentist participation in the two programs. Variables drawn from IDTS data included dentist age, gender, and practice location. We did not include race/ethnicity due to the high rate of respondents with unknown racial/ethnic status (*n* = 50, 9.2%). Practice location was classified at the county level using the U.S. Department of Agriculture’s 2013 Rural-Urban Continuum Codes (RUCC), which are determined by population size and adjacency to a metro area [13]. Codes 1–3 indicate urban counties, and 4–9 indicate rural counties.

We used survey responses to describe dentists’ role in the practice, practice busyness, and full- or part-time practice. Dentist role in the practice was dichotomized based on ownership: solo owner or partner vs. associate, independent contractor, or employee. Practice busyness was determined by the question: “How would you best describe your practice during the past 12 months?” Response options included “too busy to treat all requesting appointments,” “provided care to all requesting it, but felt overworked,” “provided care to all requesting it, but did not feel overworked,” and “not busy enough, would have liked more patients” [14]. Dentists who selected the first 2 categories were coded as *too busy*, those in the third category were coded *just busy enough* and those in the final category were coded *not busy enough*. Full-time practice was defined as practicing 32 or more hours per week.

### Statistical analyses

We performed descriptive, bivariate, and binary logistic regression analyses to address our research questions. Bivariate analyses included χ^2^ and *t* tests to examine associations between demographic and practice characteristics and participation in each program. Separate binary logistic regression models were run for the 2 dependent variables, and all independent variables were entered as a block. An interaction term including gender and age was initially included but was not statistically significant so was excluded in the final model. The Hosmer–Lemeshow goodness of fit test was used to evaluate model fit, and overall model χ^2^ tests were used to determine predictive power. Statistical significance was determined using a *P* value cutoff of 0.05. Analyses were conducted using IBM SPSS Version 23.

## Results

**Fig. 1:**
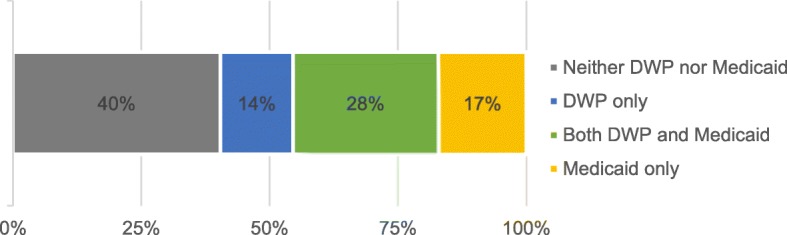
Proportion of Iowa private practice dentists participating in DWP and/or Medicaid, 2016 (*n* = 544)

A total of 544 dentists completed the survey, for a response rate of 47% after adjusting for ineligibles. Non-response bias has been previously reported; respondents were significantly more likely to be older (51 years vs. 49 years, respectively, *P* = .004) and in solo practice (*P* = .002) compared to nonrespondents [15]. Among survey respondents, 90% (*n* = 491) were general dentists and 10% (*n* = 53) were specialists. A total of 43% (*n* = 231) of dentists were accepting new DWP patients and 45% (*n* = 246) were accepting new Medicaid patients, with 28% (*n* = 152) participating in both plans (Fig. 1). The proportion of responding dentists who were accepting new DWP patients (43%) was considerably lower than the proportion who indicated that they signed up to be a DWP provider (57%, *n* = 308).

**Fig. 2:**
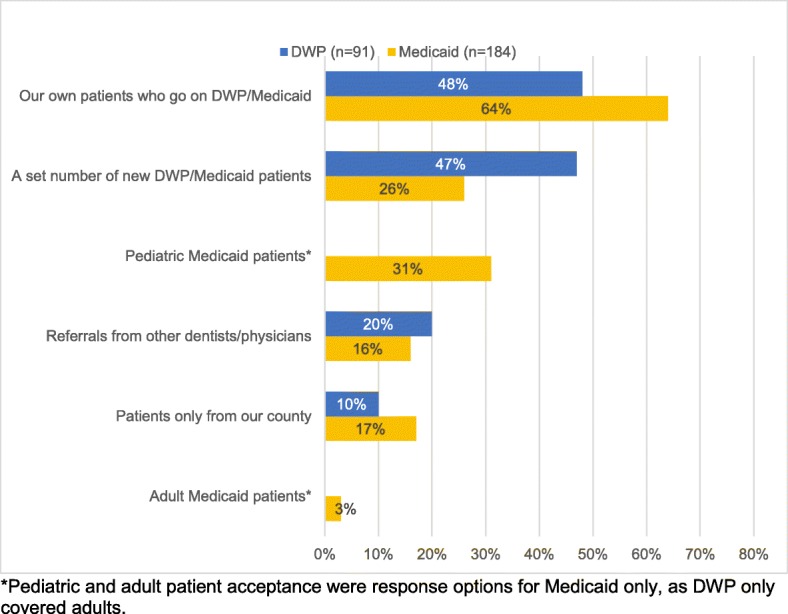
Iowa private practice dentists who participate and limit patient acceptance in DWP and Medicaid, by type of patient acceptance limitation

Participants in each program were asked whether they accept all new DWP and/or Medicaid patients or whether they place limits on patient acceptance. Among DWP-participating dentists, 60% (*n* = 139) accepted all new DWP patients and 40% (*n* = 92) placed limits on DWP patient acceptance. Among Medicaid-participating dentists, 24% (*n* = 61) accepted all new Medicaid patients and 76% (*n* = 185) placed limits on Medicaid patient acceptance. The most common limits on acceptance were accepting their own patients who transition to DWP/Medicaid, accepting a set number of new DWP/Medicaid patients, and accepting pediatric Medicaid only (Fig. 2).

Table 1 presents the demographic and practice-related characteristics of dentists who participated in the survey. A majority of respondents were male, practiced full-time, were an owner in their practice, practiced in an urban county, and reported being *just busy enough* in their practice.

**Table 1:** Descriptive demographic and practice information about Iowa private practice dentist survey respondents (*n* = 544)

Characteristic	N (%)
Age (mean)	51.7
Gender	
Female	154 (28.3)
Male	390 (71.7)
Full- vs. part-time practice	
Full time (32+ hours/week)	457 (84.3)
Part time (<32 hours/week)	85 (15.7)
Role in practice	
Sole or part owner	428 (82.1)
Associate, independent contractor, or employee	93 (17.9)
Practice busyness	
Too busy	167 (32.2)
Just busy enough	291 (56.2)
Not busy enough	60 (11.6)
Practice location	
Rural county	226 (41.5)
Urban county	318 (58.5)

In bivariate analyses, DWP participation, defined as acceptance of new DWP patients, was significantly associated with gender and practice busyness (Table 2). Male dentists were significantly more likely to participate in DWP compared to female dentists, and dentists who reported being *not busy enough* were significantly more likely to participate compared to those who reported being *just busy enough* or *too busy*. Medicaid participation, however, was only significantly associated with practice location; rural dentists were significantly more likely to participate in Medicaid compared to dentists in urban areas.

**Table 2:** Bivariate associations between DWP and Medicaid participation and demographic and practice characteristics (*n* = 544)

Characteristic	DWP Participation N(%)			Medicaid participation N(%)		
	Yes	No	*P* value	Yes	No	*P* value
Age (mean; T-test)	51.7	50.2	1.0	50.7	51.0	.70
Gender			.02			.31
Female	53 (34.4)	101 (65.6)		75 (48.7)	79 (51.3)	
Male	178 (45.6)	212 (54.4)		171 (43.8)	219 (56.2)	
Full vs. part-time practice			.31			.07
Full time (32+ hours/week)	199 (43.5)	258 (56.5)		215 (47.0)	242 (53.0)	
Part time (<32 hours/week)	32 (37.6)	53 (62.4)		31 (36.5)	54 (63.5)	
Role in practice			.26			.83
Solo owner or partner	193 (44.2)	244 (55.8)		197 (45.1)	240 (54.9)	
Associate, independent contractor, or employee	36 (37.9)	59 (62.1)		44 (46.3)	51 (53.7)	
Practice busyness			.01			.75
Too busy	69 (41.3)	98 (58.7)		74 (44.3)	93 (55.7)	
Just busy enough	119 (40.9)	172 (59.1)		139 (47.8)	152 (52.2)	
Not busy enough	37 (61.7)	23 (38.3)		29 (48.3)	31 (51.7)	
Practice location			.11			.002
Rural county	105 (46.5)	121 (53.5)		120 (53.1)	106 (46.9)	
Urban county	126 (39.6)	192 (60.4)		126 (39.6)	192 (60.4)	

In the final logistic regression models, factors predicting DWP or Medicaid participation differed (Table 3). DWP participation was significantly associated with practice busyness; dentists who reported being *not busy enough* were more than twice as likely to participate in DWP compared to those who were *too busy* or *just busy enough*. However, practice busyness was not a significant predictor of Medicaid participation after adjusting for other covariates. Rather, the only significant predictor of Medicaid participation was practice location; dentists in rural areas were almost twice as likely to participate in Medicaid compared to dentists in urban areas.

**Table 3:** Binary logistic regression models predicting dentist participation in DWP and in Medicaid (*n* = 512)

	DWP		Medicaid	
Variable	OR (95% CI)	*P* value	OR (95% CI)	*P* value
Age	1.01 (.99–1.02)	.43	1.01 (.99–1.03)	.26
Gender				
Male	1.44 (.92–2.23)	.11	.73 (.48–1.13)	.16
Female (ref)	1.00		1.00	
Full vs. part-time practice				
Full time (32+ hours/week)	1.15 (.68–1.95)	.60	1.45 (.86–2.44)	.16
Part time (<32 hours/week) (ref)	1.00		1.00	
Role in practice				
Solo owner or partner	1.14 (.69–1.89)	.61	.94 (.58–1.54)	.82
Associate, independent contractor, or employee (ref)	1.00		1.00	
Practice busyness				
Too busy	.40 (.21–.75)	.004	.73 (.39-	.31
Just busy enough	.42 (.23–.75)	.003	1.34).90 (.51–1.58)	.70
Not busy enough (ref)	1.00		1.00	
Practice location				
Rural county (ref)	1.00		1.00	
Urban county	.70 (.48–1.00)	.051	.53 (.37–.77)	.001

## Discussion

In this cross-sectional study of dentist participation in 2 separate public dental benefit programs in Iowa, we found important differences in participation patterns and factors associated with participation between the 2 programs. Despite attempts to overcome traditional barriers to dentist participation in Medicaid (e.g., higher reimbursement, interaction with a private dental carrier), the overall proportion of Iowa dentists participating in DWP 2 years after program implementation was slightly less than the proportion participating in Medicaid (43% in DWP versus 45% in Medicaid). However, Medicaid-participating dentists were much more likely than DWP-participating dentists to place limits on patient acceptance (76% versus 40%, respectively). A prior analysis found that among dentists not participating in DWP, reimbursement rate was the biggest barrier to participation [15].

While there was considerable overlap in dentist participation between the 2 programs, some dentists chose to participate in one program and not the other. The differences in factors that predicted participation between the programs suggest potentially different motivations for participation. As DWP was a relatively new program with higher reimbursement, dentists’ decision to participate may have been geared toward increased productivity, which is consistent with our identified association between DWP participation and busyness. On the other hand, traditional Medicaid was an established, state-run program with lower reimbursement; therefore, motivations to participate may be more related to altruism and, in the case of rural dentists, a desire to take care of patients in their local community if there were few dentists in the area [8]. Additionally, almost one-third of Medicaid participating dentists limited patient acceptance to children only; therefore, motivations may be driven by the desire to provide care to low-income children, whereas DWP included only adults.

Our findings concur with some previous studies and contrast with others. Our results are consistent with studies in both Iowa and California that found rural practice location to be a significant positive predictor of Medicaid participation [8, 16]. Since most other studies of dentist participation in Medicaid have not included practice urbanicity as a key independent variable, future research is needed to determine whether this relationship is similar in other states. This is a particularly important issue as some states have documented a declining number of dentists practicing in rural areas, which could exacerbate barriers to dental care among rural Medicaid enrollees [17].

In contrast to our findings, one study examining Medicaid participation by Florida dentists found that participants tended to be busier than non-participants. However, the Florida study was limited to primary care dentists who treated children, as Florida Medicaid did not include adult dental benefits at the time [14]. Also in contrast to our results, a separate Florida study found that female dentists were more likely than male dentists to participate in Medicaid, whereas we did not find gender to be a significant predictor of Medicaid or DWP participation in the final models [18].

There are several limitations to this study. First, we could not examine the role of race or ethnicity in Medicaid participation due to the low response variance and high amount of missing data on race/ethnicity among Iowa dentists. Previous studies have found that non-white dentists are more likely to participate in Medicaid than white dentists; however, we were not able to assess this in our study [7, 19]. Additionally, there are other factors that have been found to influence dentist Medicaid participation that we were not able to assess in this study, including levels of dentist altruism [8].

Non-response bias may be considered a limitation in this study. A previous assessment of non-response bias for this survey found that respondents were younger and more likely to be in solo practice than non-respondents. As age was not a significant predictor of participation in either plan, it is unlikely that this influenced the generalizability of our results. However, we were not able to examine the role of practice structure in this study, therefore it is not clear whether non-response bias with regard to solo vs. group practice impacts study generalizability.

An additional limitation relates to how dentist participation is defined. In this study we define dentist participation as acceptance of new patients. However, this study did not include the degree of participation (e.g., the number or proportion of DWP or Medicaid patients in dental practices) due to concerns regarding accuracy of self-report data for this type of item and comparability across different practice sizes. This can be considered a missing element in the discussion about the degree of dentist participation in DWP and Medicaid, and future research using insurance claims data could be used to assess this concern.

Although excluding the degree of participation can be considered a limitation in this study, we consider the acceptance of new patients to be an important measure of Medicaid enrollees’ access to dental care, and we note that the importance of this measure is also supported in research and national surveillance of physician participation in Medicaid [20, 21]. There is considerable variation in the literature regarding how Medicaid provider participation is defined, including whether dentist enrollment as a Medicaid provider is a valid measure of dentist participation [22, 23]. Our analysis found a considerable discrepancy between the proportion of dentists who were enrolled as DWP providers (57%) and those who reported actually accepting new DWP patients (43%). As the main concern is DWP enrollees’ ability to find a dentist who accepts their insurance, the latter statistic provides a more accurate representation of access.

## Conclusions and policy implications

Dentist participation in Medicaid is an ongoing concern for states aiming to ensure access to dental care for low-income populations. This study compared dentist participation in the Dental Wellness Plan – a plan for the Medicaid expansion population – with participation in traditional Medicaid. We found that while overall dentist participation in DWP was slightly less than in traditional Medicaid 2 years after DWP implementation, Medicaid participants were considerably more likely than DWP participants to place limits on new patient acceptance. We found distinct participation predictors between a traditional Medicaid dental program and the DWP, suggesting different motivations for participation in the two programs. State Medicaid programs may consider stronger provider outreach to target motivating factors for participation. This study contributes to a body of research to better understand the impact of program- and policy-related factors on dentist Medicaid participation.

## Additional file

Additional file 1: Survey instrument. Description of data: Survey instrument fielded to all Iowa private practice dentists in October 2016 (PDF 1020 kb)

## Abbreviations

ACA: Affordable Care Act
DWP: Dental Wellness Plan
FPL: Federal Poverty Level
IDTS: Iowa Dentist Tracking System
RUCC: Rural-Urban Continuum Codes
UCR: Usual, customary, and reasonable
