# Supplementary material for: A tale of two public dental benefit programs: Iowa dentist participation in traditional Medicaid versus a Medicaid expansion program
Source: BMC Oral Health. 2019 May 24;19:89. doi: 10.1186/s12903-019-0771-z (PMC6534867; doi:10.1186/s12903-019-0771-z)
Supplement: Supplementary file 1 — Survey instrument. Description of data: Survey instrument fielded to all Iowa private practice dentists in October 2016 (PDF 1020 kb) [file 12903_2019_771_MOESM1_ESM.pdf]

The University of Iowa
Public Policy Center

Iowa Dental Wellness Plan Survey

Survey instructions: Answer each question by marking the box to the left of your answer.

You are sometimes told to skip over some questions in this survey. When this happens you will see an arrow with a note that tells you what question to answer next, like this:

- ☐ Yes
- ☐ No ➔ If No, Go to #4

If you make a mistake, please **cross out** the incorrect answer and **circle** the correct answer. If there is a question that you are uncomfortable answering, feel free to skip to the next question. If you have questions, please call 1-800-710-8891.

If you practice in more than one location, please answer the questions in this survey as they pertain to what you consider your *primary practice location.*

1. Have you signed up to be a Dental Wellness Plan (DWP) provider?

   1☐ Yes ➔ **Go to #5 (p. 3)**
   2☐ No ➔ **Go to #2 (below)**

2. Why did you choose not to sign up for the DWP? Please **write "X" next to the three most important reasons** you chose not to sign up.

   ____ Reimbursement rates
   ____ Administered by Delta Dental
   ____ Scope of covered services
   ____ Busyness of my practice
   ____ DWP patient-related reasons
   ____ DWP is a new program
   ____ My practice could be the only one in the area accepting DWP
   ____ My practice participates in Title 19 instead
   ____ Other, *please describe:*________________________________

   ☐ Not sure/I am not responsible for this decision

First Mailing

3. How likely are you to sign up to be a DWP provider within the next year?

1☐ Extremely likely
2☐ Moderately likely
3☐ Slightly likely
4☐ Not at all likely

5☐ Not sure/I am not responsible for this decision

4. Could any change be made to the DWP program that would increase the likelihood of you signing up?

1☐ Yes, *please describe:* ____
2☐ No

Go to Question 6 (p.4)

5. Do you currently accept NEW Dental Wellness Plan patients into your practice?

1☐ YES

*If you **are currently accepting** **new** **DWP patients**, please answer **a-b** below.*

a. Do you accept all DWP members who contact you as new patients?

1☐ Yes

2☐ No, in our office we only accept some new DWP including:
Select all that apply.
- 1☐ A set number of new DWP patients
- 2☐ Our own patients who go on DWP
- 3☐ Referrals from other dentists/physicians
- 4☐ Patients only from our county
- 5☐ Other: ____________________________

b. How seriously have you/your practice considered stopping your acceptance of new DWP patients since the program began?

1☐ Extremely seriously
2☐ Moderately seriously
3☐ Slightly seriously
4☐ Not at all seriously

5☐ Not sure/I am not responsible for this decision

2☐ NO

*If you **are not currently accepting** **new** **DWP patients**, please answer **a-b** below.*

a. Did you ever accept new DWP patients?

1☐ Yes, I did at one time but have stopped accepting new DWP patients

2☐ No, I have never accepted DWP patients

b. Why did you choose not to accept new DWP patients? Please **write "X" next to the three most important reasons** you chose not to accept new DWP patients.

____ Reimbursement rates

____ Administered by Delta Dental

____ Scope of covered services

____ Busyness of my practice

____ DWP patient-related reasons

____ DWP is a new program

____ My practice could be the only one in the area accepting DWP

____ My practice participates in Title 19 instead

____ Other, *please describe:*

____

☐ Not sure/I am not responsible for this decision

6. ***About*** what percentage of your current patients are covered by the Dental Wellness Plan?

☐☐☐ %

7. Where do you ***primarily*** refer Dental Wellness Plan patients who you are not interested in accepting or able to accept in your practice? *Please select only one.*

1☐ I accept them all
2☐ Community Health Center
3☐ The UI College of Dentistry
4☐ Another local practice
5☐ DWP's "Find a Dentist" website
6☐ I don't have a good place to refer
7☐ Other: ______________________

8. Who was ***primarily*** responsible for making the decision whether your practice would accept Dental Wellness Plan patients? *Please select only one.*

1☐ I was
2☐ The dentists in the practice as a group
3☐ The owner of the practice
4☐ The clinic management/administration
5☐ Other: _________________________

9. Which best describes your overall attitude toward the Dental Wellness Plan?

1☐ Very positive
2☐ Somewhat positive
3☐ Somewhat negative
4☐ Very negative
5☐ Not sure/Don't know

10. Please read the following statements about the Dental Wellness Plan (DWP) and circle the number that indicates the degree to which you disagree or agree with these statements.

	Strongly disagree	*Disagree*	*Agree*	*Strongly agree*	*Not sure/ Don't know*
a. Without the DWP program, these low income patients would not be able to get adequate dental care	**1**	**2**	**3**	**4**	**NS**
b. The DWP program respects dentists' professional judgment concerning patient care	**1**	**2**	**3**	**4**	**NS**
c. Dentists can have an impact on the policies of the DWP program	**1**	**2**	**3**	**4**	**NS**

EARNED BENEFITS APPROACH

For those unfamiliar with the Dental Wellness Plan benefits: The DWP uses an *earned benefits approach*. Members can earn additional covered benefits by going to the dentist for regular exams. There are three levels of coverage:

i. Core Benefits are available after a patient's first dental exam. This level includes diagnostic, preventive, emergency, and stabilization services.
ii. Enhanced Benefits are available if a patient returns for a second exam 6-12 months after the first. This level includes Core Benefits plus routine restorative, endodontic care, and extractions.
iii. Enhanced Plus Benefits are available if a patient returns for a third exam 6-12 months after the second, and as long as the patient returns every 6-12 months for recall exams. This level covers Enhanced Benefits plus crowns, full and partial dentures.

Members who do not return for recall exams every 6-12 months will only be eligible for Core Benefits.

11. Please indicate the degree to which you agree or disagree with the following statements about the Dental Wellness Plan earned benefits approach.

	Strongly disagree	*Disagree*	*Agree*	*Strongly agree*	*Not sure/ Don't know*
a. The earned benefits approach is an effective way to reward people who return for regular checkups	**1**	**2**	**3**	**4**	**NS**
b. The earned benefits approach makes it difficult to provide comprehensive treatment to DWP patients	**1**	**2**	**3**	**4**	**NS**
c. The earned benefits approach will increase the likelihood that patients return for regular exams	**1**	**2**	**3**	**4**	**NS**
d. The earned benefits approach prevents DWP patients from getting the care they need when they need it	**1**	**2**	**3**	**4**	**NS**
e. The earned benefits approach will increase the likelihood that patients take better care of their oral health	**1**	**2**	**3**	**4**	**NS**

12. Which best describes your attitude toward the earned benefits approach?

1☐ Very positive
2☐ Somewhat positive
3☐ Somewhat negative
4☐ Very negative

5☐ Not sure/Don't know

YOUR EXPERIENCES WITH THE DENTAL WELLNESS PLAN

*** If you have not signed up for the Dental Wellness Plan, please go to **question 23 (p. 7)** ***

13. Which best describes your attitude toward Delta Dental's administration of the Dental Wellness Plan?

- 1☐ Very positive
- 2☐ Somewhat positive
- 3☐ Somewhat negative
- 4☐ Very negative
- 5☐ Not sure/Don't know

14. Have you had difficulty referring your DWP patients to any dental specialists?

- 1☐ Yes
- 2☐ No ➔ **Go to #16**
- 3☐ N/A – I am a specialist ➔ **Go to #16**

15. Which types of dental specialists have you had difficulty referring your DWP patients to? *Select all that apply.*

- ☐ Oral surgeon
- ☐ Periodontist
- ☐ Endodontist
- ☐ Prosthodontist

16. How satisfied are you with the Dental Wellness Plan overall?

- 1☐ Very satisfied
- 2☐ Satisfied
- 3☐ Dissatisfied
- 4☐ Very dissatisfied

17. Would you recommend DWP participation to other Iowa dentists?

- 1☐ Definitely yes
- 2☐ Probably yes
- 3☐ Probably no
- 4☐ Definitely no

18. Starting July 2016, a second dental benefits carrier – called MCNA Dental - joined the Dental Wellness Plan in addition to Delta Dental. Have you contracted with MCNA as a participating provider?

- 1☐ Yes
- 2☐ No, but I plan to
- 3☐ No, and I do not plan to; *please describe why*: ____

PATIENT RISK ASSESSMENT

Dentists participating in DWP are encouraged to use PreViser, an online risk assessment tool. When general dentists use PreViser for their DWP patients, they receive reimbursement and are eligible for the DWP annual bonus program.

19. Do you currently use the PreViser risk assessment with any of your DWP patients?

1☐ YES

a. What is the most important reason you choose to use the PreViser risk assessment?

- 1☐ Reimbursement for completing it
- 2☐ Time needed to complete it
- 3☐ Degree of helpfulness for patient care
- 4☐ Electronic submission
- 5☐ Bonus pool program
- 6☐ Other: ______________________________
- 7☐ Not sure/I am not responsible for this decision

Go to Question 20

2☐ NO

*If you **do not currently use the PreViser risk assessment**, please answer **a** below, and then go to Question 22.*

a. What is the most important reason you choose not to use the PreViser risk assessment?

- 1☐ Reimbursement for completing it
- 2☐ Time needed to complete it
- 3☐ Degree of helpfulness for patient care
- 4☐ Electronic submission
- 5☐ Did not know about the PreViser risk assessment
- 6☐ Other: ______________________________
- 7☐ Not sure/I am not responsible for this decision

Go to Question 23 (p. 8)

20. How helpful is the PreViser risk assessment in facilitating discussions with patients about their oral health?

- 1☐ Very helpful
- 2☐ Somewhat helpful
- 3☐ A little helpful
- 4☐ Not at all helpful

21. How helpful is the PreViser risk assessment in facilitating discussions with patients about their systemic health?

- 1☐ Very helpful
- 2☐ Somewhat helpful
- 3☐ A little helpful
- 4☐ Not at all helpful

22. Which best describes your attitude toward the PreViser risk assessment?

- 1☐ Very positive
- 2☐ Somewhat positive
- 3☐ Somewhat negative
- 4☐ Very negative

23. Not counting PreViser, do you use any other risk assessment tools in your office?

- 1☐ Yes, *please describe:*____
- 2☐ No

YOUR PARTICIPATION IN TITLE 19 (MEDICAID)

24. Do you currently accept new Title 19 patients into your practice?

- 1☐ Yes
- 2☐ No ➔ **Go to #29**

25. Do you accept all new Title 19 patients?

- 1☐ Yes, I accept all new Title 19 patients
- 2☐ No, in our office we only accept the following Title 19 patients (*Select all that apply*):
  - 1☐ A set number of new Title 19 patients
  - 2☐ Our own patients who go on Title 19
  - 3☐ Adult Title 19 patients only
  - 4☐ Pediatric Title 19 only
  - 5☐ Referrals from other dentists/physicians
  - 6☐ Patients only from our county
  - 7☐ Other: ___________________________

26. How has your opinion or experience regarding the DWP changed your acceptance of new adult Title 19 patients?

- 1☐ I accept fewer adult Title 19 patients
- 2☐ No change in our acceptance of adult Title 19 patients
- 3☐ I accept more adult Title 19 patients
- 4☐ Not sure/Don't know

27. How has your opinion or experience regarding the DWP changed your acceptance of new pediatric Title 19 patients?

- 1☐ I accept fewer pediatric Title 19 patients
- 2☐ No change in our acceptance of pediatric Title 19 patients
- 3☐ I accept pediatric adult Title 19 patients
- 4☐ Not sure/Don't know

28. Do you submit claims electronically to Medicaid?

- 1☐ Yes
- 2☐ No, *please describe why not:* ____

The following two questions show some issues that dentists may have with dental insurance plans. Please **indicate how much you think that issue is a problem in the Dental Wellness Plan.**

Then, please **circle whether you think the Dental Wellness Plan is better, same, or worse compared to Title 19** on each issue.

29. Administration-related issues:

	DENTAL WELLNESS PLAN				TITLE 19			
	No problem	Minor problem	Major problem	Not sure/ Don't know	DWP is ______ than Title 19			Not sure/ Don't know
a. Time spent on paperwork	**1**	**2**	**3**	**NS**	Better	Same	Worse	**NS**
b. Denial of payment	**1**	**2**	**3**	**NS**	Better	Same	Worse	**NS**
c. Slow payment	**1**	**2**	**3**	**NS**	Better	Same	Worse	**NS**
d. Reimbursement rate	**1**	**2**	**3**	**NS**	Better	Same	Worse	**NS**
e. Intermittent eligibility	**1**	**2**	**3**	**NS**	Better	Same	Worse	**NS**
f. Difficulty of eligibility determination	**1**	**2**	**3**	**NS**	Better	Same	Worse	**NS**
g. Limited services covered	**1**	**2**	**3**	**NS**	Better	Same	Worse	**NS**

30. Patient-related issues:

	DENTAL WELLNESS PLAN				TITLE 19			
	No problem	Minor problem	Major problem	Not sure/ Don't know	DWP is ______ than Title 19			Not sure/ Don't know
a. Broken appointments	**1**	**2**	**3**	**NS**	Better	Same	Worse	**NS**
b. Complexity of patient medical history	**1**	**2**	**3**	**NS**	Better	Same	Worse	**NS**
c. Complexity of patient dental treatment needs	**1**	**2**	**3**	**NS**	Better	Same	Worse	**NS**
d. Patient non-compliance with recommended treatment	**1**	**2**	**3**	**NS**	Better	Same	Worse	**NS**
e. Not enough general dentists in the area accepting patients with the plan	**1**	**2**	**3**	**NS**	Better	Same	Worse	**NS**
f. Ability to refer to dental specialists	**1**	**2**	**3**	**NS**	Better	Same	Worse	**NS**

PRACTICE SETTING

Finally, we would like to ask some questions about your practice setting to identify how different practice characteristics relate to Iowa dentists' impressions of the Dental Wellness Plan.

31. How would you best describe your practice during the past 12 months?

- 1☐ Too busy to treat all requesting appointments
- 2☐ Provided care to all requesting it, but felt overworked
- 3☐ Provided care to all requesting it, but did not feel overworked
- 4☐ Not busy enough, would have like more patients
- 5☐ Practice limited, no new patients taken

32. In your practice, do you usually work 32 hours or more per week?

- 1☐ Yes
- 2☐ No

33. In your primary practice, do you use an electronic health record system for patient records?

- 1☐ Yes
- 2☐ No

34. How would you describe your role in your primary practice?

- 1☐ Solo practice owner
- 2☐ Partner
- 3☐ Associate buying into the practice
- 4☐ Associate not buying into the practice
- 5☐ Independent contractor
- 6☐ Employee in a corporate owned practice (e.g., Aspen, Ocean Dental, Applewhite Dental)
- 7☐ Other: ______________________

35. Please indicate your **personal gross production** in the practice last year (excluding investment or non-practice income).

- 1☐ under $200,000
- 2☐ $200,000 – $299,999
- 3☐ $300,000 – $399,999
- 4☐ $400,000 – $499,999
- 5☐ $500,000 – $599,999
- 6☐ $600,000 – $699,999
- 7☐ $700,000 – $799,999
- 8☐ $800,000 – $899,999
- 9☐ $900,000 – $999,999
- 10☐ over $1,000,000

36. What is the most important change that could be made to improve the Dental Wellness Plan?

37. We are interested in any other comments you may have about the Dental Wellness Plan.

Thank you for completing this questionnaire.
Please return it in the enclosed postage-paid envelope.
